# miRNA and lncRNA Expression Networks Modulate Cell Cycle and DNA Repair Inhibition in Senescent Prostate Cells

**DOI:** 10.3390/genes13020208

**Published:** 2022-01-24

**Authors:** Willian A. da Silveira, Ludivine Renaud, Edward S. Hazard, Gary Hardiman

**Affiliations:** 1 Department of Biological Sciences, Science Centre, School of Health, Science and Wellbeing, Staffordshire University, Leek Road, Stoke-on-Trent ST4 2DF, UK; willian.dasilveira@staffs.ac.uk; 2 Faculty of Medicine, Health and Life Sciences, Institute for Global Food Security (IGFS), School of Biological Sciences, Queen’s University Belfast, 19 Chlorine Gardens, Belfast BT9 5DL, UK; 3 Department of Medicine, Medical University of South Carolina, MSC 403, 171 Ashley Ave Suite 419, Charleston, SC 29425, USA; renaudl@musc.edu (L.R.); hazardes3@gmail.com (E.S.H.)

**Keywords:** ncRNA, miRNA, lncRNA, cell senescence, prostate cell, cell cycle, DNA repair, transcriptomics, network biology

## Abstract

Cellular senescence is a state of permanent growth arrest that arises once cells reach the limit of their proliferative capacity. It creates an inflammatory microenvironment favouring the initiation and progression of various age-related diseases, including prostate cancer. Non-coding RNAs (ncRNAs) have emerged as important regulators of cellular gene expression. Nonetheless, very little is known about the interplay of microRNAs (miRNAs) and long non-coding RNAs (lncRNAs) and how deregulation of ncRNA networks promotes cellular senescence. To investigate this, human prostate epithelial cells were cultured through different passages until senescent, and their RNA was extracted and sequenced using RNA sequencing (RNAseq) and microRNA sequencing (miRNA-seq) miRNAseq. Differential expression (DE) gene analysis was performed to compare senescent and proliferating cells with Limma, miRNA-target interactions with multiMiR, lncRNA-target interactions using TCGA data and network evaluation with miRmapper. We found that miR-335-3p, miR-543 and the lncRNAs H19 and SMIM10L2A all play central roles in the regulation of cell cycle and DNA repair processes. Expression of most genes belonging to these pathways were down-regulated by senescence. Using the concept of network centrality, we determined the top 10 miRNAs and lncRNAs, with miR-335-3p and H19 identified as the biggest hubs for miRNAs and lncRNA respectively. These ncRNAs regulate key genes belonging to pathways involved in cell senescence and prostate cancer demonstrating their central role in these processes and opening the possibility for their use as biomarkers or therapeutic targets to mitigate against prostate ageing and carcinogenesis.

## 1. Introduction

Somatic cells possess a limited proliferative capacity and after a certain number of cell divisions, they reach a state of permanent growth arrest termed cellular senescence [1]. Accumulation of senescent cells is a feature observed in aged organisms and excessive buildup of these cells in tissues can cause deleterious effects on secretion, metabolism, and regenerative capacities, creating an inflammatory microenvironment that favours the initiation and progression of various age-related diseases, including cancer [1,2]. 

The human prostate is an important gland in the male reproductive system, producing close to 30% of seminal fluid and providing nutrients for sperm [3]. Prostate cancer is the most common cancer in males [4]. This disease has age as its most significant risk factor, with 70% to 90% of men over 80 years of age harbouring undetected foci for the disease [5]. Race represents another risk factor for prostate cancer. African American men have increased prostate cancer risk and a greater mortality rate than European-American men and have higher expression of genes associated with immune response and inflammation [6].

There is growing interest in finding novel markers of senescence that could be used as prognostic markers or drug targets for both ageing and cancer [7]. Since a universal marker for senescence has yet to be discovered, a set of hallmarks describing senescence in a general context have been established [8]. However, complexities, including phenotype heterogeneity, confound the evaluation of senescence, particularly within complex tissues and living organisms [9]. To address these challenges, research programmes are targeting the discovery of novel and more specific biomarkers using optimised combinatorial strategies coupled to emerging detection techniques [9].

Senescent cells are observed in the ageing prostate and a senescence-associated secretory phenotype has been linked to benign prostatic hyperplasia (BPH) and prostate cancer [10]. Several signalling pathways provide biological credibility for the role of senescence in both BPH and prostate cancer, although proving causality is difficult. The theory of senescence as a mechanism for age-related prostate disease creates clinical implications and offers opportunities for therapeutic targeting in the future [10].

Non-coding RNAs (ncRNAs) play important roles in the ageing process [11,12]. Of the ncRNAs, microRNAs (miRNAs) are a class of single-stranded molecules consisting of 19–24 nucleotides that canonically regulate gene expression by down-regulating translation or leading to the degradation of target mRNAs [13]. Several miRNAs have been recognised to regulate ageing-associated processes such as decreased self-renewal in stem cells, activation of the p53 pathway, inhibition of cell proliferation and differentiation, and induction of cell senescence [12]. Long non-coding RNAs (lncRNAs) are functional transcripts longer than 200 nucleotides that are not translated into proteins and their regulatory role is quite diverse in nature and function [14]. LncRNAs play critical roles in mechanisms linked with ageing including proliferation, differentiation, and apoptosis. Premature ageing is linked with altered expression of lncRNAs that regulate telomere length [12]. Advances in high-throughput sequencing and computational approaches have greatly progressed our understanding of lncRNAs in the past few years [15,16]. LncRNAs could serve as biomarkers to differentiate low risk from high-risk diseases. Additionally, they may become therapeutic targets for advanced and castrate-resistant cancers. Prostate cancer-specific lncRNAs are compelling as diagnostic biomarkers owing to their high tissue and tumour specificity, and presence in bodily fluids [17]. 

Interestingly, considering the role of ncRNAs in ageing processes and tumorigenesis, a knowledge gap currently exists regarding the involvement of these molecules in the mechanism of prostate cell senescence. Our study is the first to assess transcriptomic differences between proliferating and senescent prostate cells, and to report a network of ncRNAs involved in cell cycle arrest, with the potential to be used for the development of new biomarkers and therapeutic targets against ageing and prostate cancer.

## 2. Materials and Methods

### 2.1. Primary Human Cells

Primary prostate epithelial cells (PrECs) derived from a healthy 53-year-old male with no history of prostate disease were obtained from Clonetics^TM^ (San Diego, CA, USA). The PrEC used in this study were cryopreserved in the second passage as proliferating cultures. The cells were Telomerase Reverse Transcriptase (TERT) expression negative indicative that were primary cells and not an immortalised cell line. PrECs were cultured in Clonetics™ PrEGM™ Prostate Epithelial Cell Growth Medium, with a confluence of 70–90% in 0.2 mL/well 96 well plates– and approximately 0.1 × 10^6^ cells for each well – and supplemented with 10% fetal bovine serum, 2mM glutamine, and 1% penicillin/streptomycin. Passage number for cell culture is a record of the number of times the culture has been subcultured, i.e., harvested and reseeded into multiple ‘daughter’ cell culture flasks. The population doubling (PD) number is defined as the approximate number of doublings that the cell population underwent since they were isolated. For cell passages, we used a split ratio 1:2 and cells were taken through seven population doublings (PD) until they were senesced.

### 2.2. RNA Extraction RNA Sequencing

RNA was extracted using TRIzol reagent (Invitrogen) from PrECs passage 1 (proliferative) and passage 7 (senescent) and the extracted RNA were further purified using the RNeasy Mini kit (Qiagen, Valencia, CA, USA). RNA was assessed via absorbance readings (OD) at 260 nm using an ND-1000 (Nanodrop, Wilmington, DE). RNA integrity was examined with the 6000 Nano LabChip assay from Agilent, (Santa Clara, CA, USA). Only RNA samples with an RNA integrity number (RIN) score of >7.0 indicating intact RNA were used for RNAseq experiments (RIN is a measure of the degradation of RNA sample) [18]. We used biological replicate samples to assess both mRNA and miRNAs in each sample (*n* = 2 independent cell cultures for the proliferative and senescent cells respectively). Library preparation was made using the TruSeq RNA Sample and TruSeq Small RNA Library Prep Kits (Illumina, San Diego, CA, USA) according to manufacturer instructions. Sequencing was performed on a HiSeq2500 (Illumina, San Diego, CA, USA) using a SE50 strategy. The mRNA and miRNA libraries were sequenced to a minimum depth of 25 and 5 million reads respectively. RNA-Seq data have been submitted to the NCBI Gene Expression Omnibus, accession number GSE189209.

### 2.3. Gene Expression and System-Level Analysis

OnRamp’s advanced Genomics Analysis Engine used an automated RNAseq workflow to process the data, including (1) data validation and quality control, (2) read alignment to the human genome (hg19) using STAR RNA-seq aligner, (3) generation of gene-level count data with HTSeq, and (4) differential expression analysis with DEseq2, which enabled the inference of differential signals with robust statistical power [15,19,20]. The resulting SAM files derived from the alignment step were sorted and inputted into the Python package HTSeq to generate count data for gene-level differential expression analyses [21]. Count data were analysed with Limma [22] version 3.29.11 and R version 3.3.1 [23] and a multi-factorial analysis was used to correct potential batch effects. Only transcripts with a modular linear fold change ≥1.5 and a Benjamini-Hochberg FDR adjusted *p*-value of ≤0.01 [24] were considered differentially expressed (DE). The DE genes were used as input for impact analysis using iPathwayGuide (Advaita Bioinformatics) [25].

### 2.4. miRNA and lncRNA Network Analysis

After sequencing, miRNA analysis followed the CAP-miRSeq pipeline [26]. This involved two rounds of alignments to the human genome (hg19) and counting using Bowtie/HTSeq and the miRDeep2 mapper/miRDeep2 module. Count data were analysed with Limma version 3.29.11 [22] and R version 3.3.1 [23] and a multi-factorial analysis was used to correct potential batch effects. Only miRNAs with a modular linear fold change ≥1.5 and a Benjamini-Hochberg [24] FDR adjusted *p*-value of ≤0.01 were considered differentially expressed (DE) and used in downstream analyses. The miRNA—target network was built using the R package multiMiR version 2.1 [27] followed by the use of miRmapper version 1.0 [28], a tool for identifying the most central miRNAs in biological networks, as opposed to merely ranking those miRNAs with the greatest fold change in expression levels.

The lncRNAs and their target genes were extracted from the list of DE RNA transcripts selected from the Long Noncoding RNA Heterogeneous Regulatory Network integrator (LONGHORN) algorithm predictions made using the Cancer Genome Atlas Prostate Adenocarcinoma (TCGA-PRAD) [29]. LongHorn predictions are based on reverse-engineered canonical interactions, determined experimentally as part of the Encyclopaedia of DNA Elements (ENCODE) project including the crosslinking and immunoprecipitation assay (eCLIP) and chromatin immunoprecipitation sequencing (ChIP-seq) data [29]. The centrality of lncRNAs in the networks was used to rank lncRNAs in the same manner that we ranked the miRNAs. Simply described, centrality is a measure of the degree, i.e., the number of edges connected to a vertex; the assumption being that vertices with the highest degrees (i.e., those with the most connections) play critical roles in the functioning of a system, and focusing attention on the system’s most fundamental elements. Centrality, when applied to ncRNA–mRNA interaction networks, can emphasise which players are more important than others in a specific context such as disease or biological processes by defining how many in-degrees and out-degrees each ncRNA possesses [28].

### 2.5. Expression Network Visualisation and Biological Representation

The miRNA and lncRNA expression networks were merged and the visualisations were generated using the GeneMania Force Directed Layout on Cytoscape version 3.7.2 [30] and GeneMania version 3.5.2 [31]. Only DE genes contained in the Gene Ontology Biological Process Cell Cycle (GO:0007049) and Cell Proliferation (GO:0008283) terms were presented to facilitate interpretation. For network presentation, up-regulated genes were depicted as red, down-regulated genes as blue, miRNAs as triangles, lncRNAs as octagons and genes as rectangles respectively.

The impacted genes obtained from the KEGG Prostate Cancer (hsa05215) iPathwayGuide Advaita Bioinformatics [25] analyses were overlapped with the representation of the DE miRNAs, DE lncRNAs and DE genes in the pathway. The signalling pathway impact analysis (SPIA) [32] is a topology-based pathway analysis approach that identifies and predicts which genes belonging to the pathway have perturbed function when the DE genes are considered. Overrepresentation analysis of the genes present in the network was performed using the webtool ToppFun and the Gene Ontology Cell Component database [33,34,35].

## 3. Results

### 3.1. Comparison of Senescent and Proliferating Cells Revealed miRNA-335-3p and lncRNA H19 as the Central Altered ncRNAs

**Figure 1:**
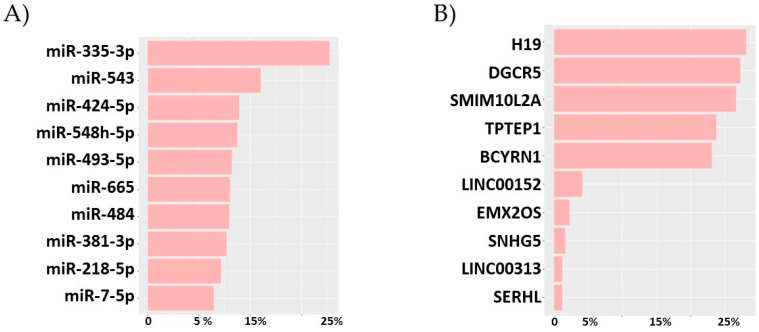
Top 10 non-coding RNAs altered by prostate cell senescence classified by network centrality. (**A**) miRNAs, (**B**) lncRNAs. Percentage is related to the total number of DE targets. DE ncRNA threshold in limma was log 2 Fold Change ≥ |1| and adjusted *p*-value (FDR) < 0.01. Network Analysis was performed with miRmapper.

DE analysis of total RNA uncovered 1361 DE genes in the comparison “late vs. early” passages of cell culture (Supplemental Table S1). Pathway impact analysis found that these genes mapped to gene ontology (GO) terms related to the cell cycle (Table 1 and Supplemental Table S2). DE analysis of miRNAs uncovered 97 DE miRNAs in late passage cells (Supplemental Table S3). The miRNA-gene target network revealed *miRNA-335-3-p*, *miRNA-543*, *miRNA-424-5p*, *miRNA-548h-5p*, *miRNA-493-5p*, *miRNA-665*, *miRNA-484*, *miRNA-381-3p*, *miRNA-218-5p* and *miRNA-7-5p* as the Top 10 hub miRNAs based on their high degree of centrality (Figure 1A, Supplemental Table S4). DE analysis recognised 13 DE lncRNAs from the senescent PrECs (Supplemental Table S5). Of these *H19*, *DGCR5*, *SMIM10L2A*, *TPTEP1*, *BCYRN1* possessed more than 100 DE gene targets, where the rest possessed lower than 20 expressed targets, Figure 1B and Table S6.

### 3.2. Senescent Prostate Cells miRNAs and lncRNAs Control the Cell Cycle and Proliferation by Regulating a Complementary Set of mRNA Targets

Of the 1361 DE genes uncovered, 828 (60.8%) are predicted to be regulated by the top 10 miRNAs and/or lncRNAs (Table S7), with the top miRNAs regulating 567 targets (68.5%) of the ncRNA-gene target network and 41.7% of all the DE genes, and the top lncRNAs regulating 478 targets (57.7%) of the network and 35.1% of all DE genes.

**Figure 2:**
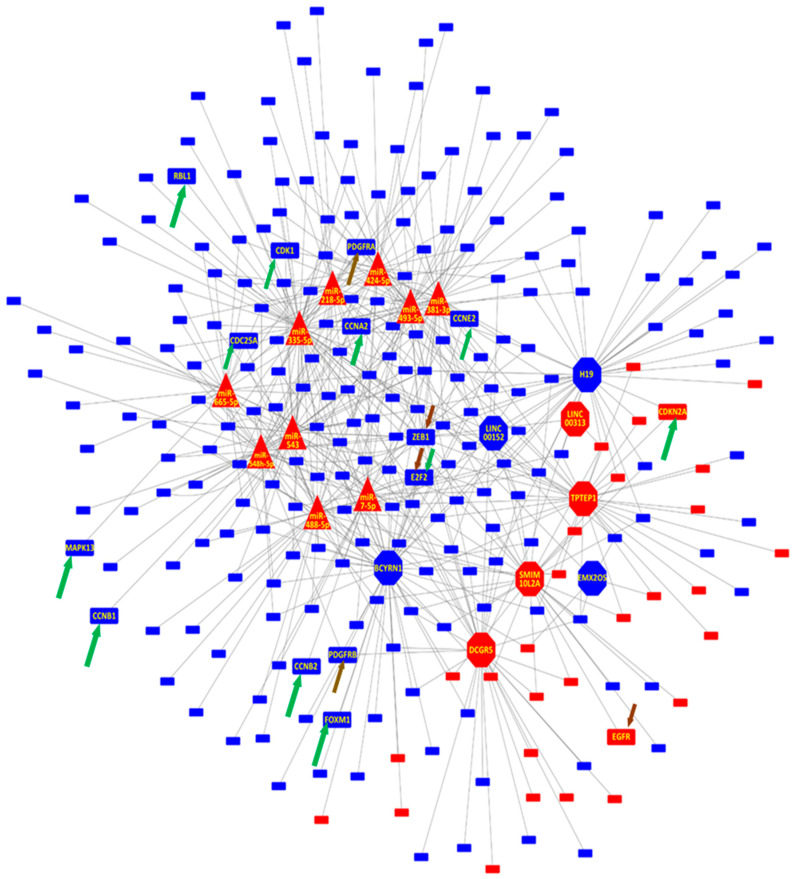
Network representation of lncRNAs and miRNAs gene targets for prostate cell senescence. Only genes related to Cell Cycle and Cell Proliferation are presented. Gene Targets *PDGFRB, PDGFRA, CCNE2, E2F2, EGFR, ZEB1* belonging to the Prostate Cancer KEGG pathway are highlighted (gold arrow) as are *CCNB1, CCNB2, FOXM1, CCNA2, CDK1, CCNE2, CDC25A, E2F2, CDKN2A, MAPK13, RBL1* (green arrow) from the Cellular Senescence pathway. The lncRNAs *MYBL, SERHL* and *SNHG5* are not depicted as they do not have any gene targets belonging to Cell Cycle and/or Cell Proliferation. Blue indicates down-regulated and red up-regulated in senescent cells relative to non-senescent cells. Genes are presented as rectangles, miRNAs as triangles, and lncRNAs as octagons.

Interestingly, although both miRNAs and lncRNAs are involved in the regulation of the Cell Cycle, only 217 (26% of the whole network and 15.9% of the DE genes) are co- regulated by both an miRNA and lncRNA. An overrepresentation analysis of the three sets of gene targets, the overlap between the miRNA and lncRNA targets, and the unique sets for each regulatory RNA category, show enrichment of terms related to the kinetochore, centriole and centrosome in all the gene lists (Table S8). This indicates that miRNAs and lncRNAs are impacting the down-regulation of the cell cycle, acting on the same cellular components but with their functions complementing each other as they regulated both a shared and a unique set of genes related to the same terms (Figure 2 and Table S7).

In addition, we encountered 11 DE genes belonging to the KEGG Senescence pathway (*CCNB1*, *CCNB2*, *FOXM1*, *CCNA2*, *CDK1*, *CCNE2*, *CDC25A*, *E2F2*, *CDKN2A*, *MAPK13, RBL1*) and 6 DE genes from the Prostate Cancer Pathway (*PDGFRB*, *PDGFRA*, *CCNE2*, *E2F2*, *EGFR*, *ZEB1*), with *CCNE2* and *E2F2* belonging to both. Of these genes, *ZEB1* is the gene being regulated by the greatest number of miRNAs (miR-335-3p, miR-493-5p, miR-543 and miR-548h-5p) and LncRNAs (*H19*, *LINC00313*, *LINC00152*, *TPTEP1, LINC00086/SMIM10L2A* and *DGCR5*), (Figure 2).

### 3.3. Prostate Cell Senescence Induced DNA Repair Gene Down-Regulation through ncRNA Network

**Figure 3:**
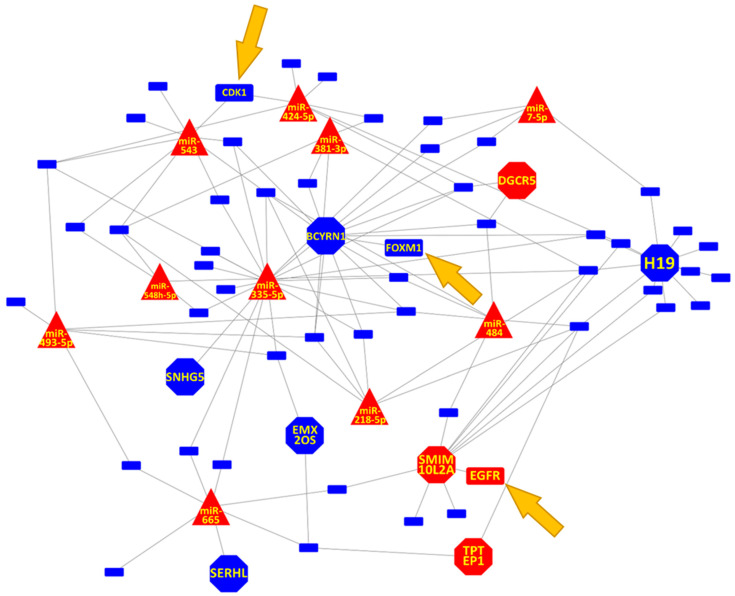
Network representation of lncRNAs and miRNAs gene targets for prostate cell senescence. Only genes related to DNA Repair are presented. The gene target *EGFR* (belonging to the Prostate Cancer KEGG pathway) is highlighted as is *FOXM1, CDK1*, (from the Cellular Senescence pathway). The lncRNAs *LINC00152* and *LINC00313* are not depicted as they do not have any gene target mapping to either the Prostate Cancer and Cellular Senescence pathways. Blue indicates down-regulated and red up-regulated in senescent cells relative to non senescent cells. Genes are presented as rectangles, miRNAs as triangles, and lncRNAs as octagons. *EGFR*, *FOXM1*, *CDK1* are indicated with arrows.

**Figure 4:**
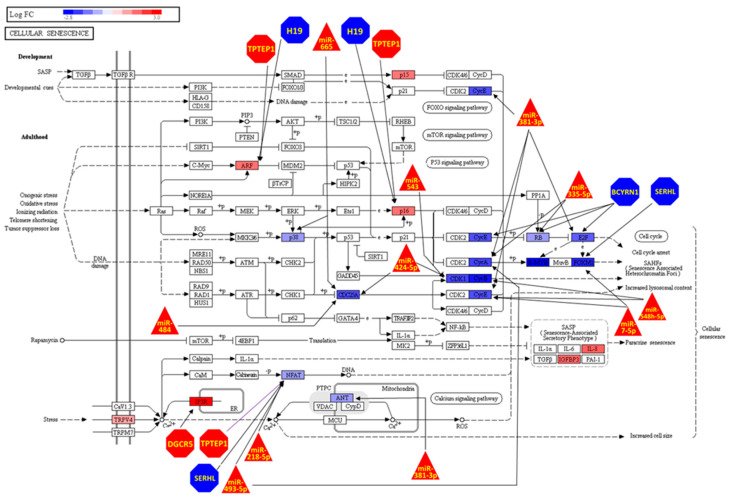
Cell Senescence KEEG pathway genes being regulated by ncRNAs due Cell Senescence. The transcription factor *NFAT1* is the target of many ncRNAs. DE RNAs threshold in limma was log 2 Fold Change ≥ |1| and adjusted *p*-value (FDR) < 0.01. KEGG Pathway depiction was generated using Advaita iPathwayGuide. Blue indicates down-regulated and red up-regulated in senescent cells relative to non senscent cells. Genes are presented as rectangles, miRNAs as triangles, and lncRNAs as octagons.

**Figure 5:**
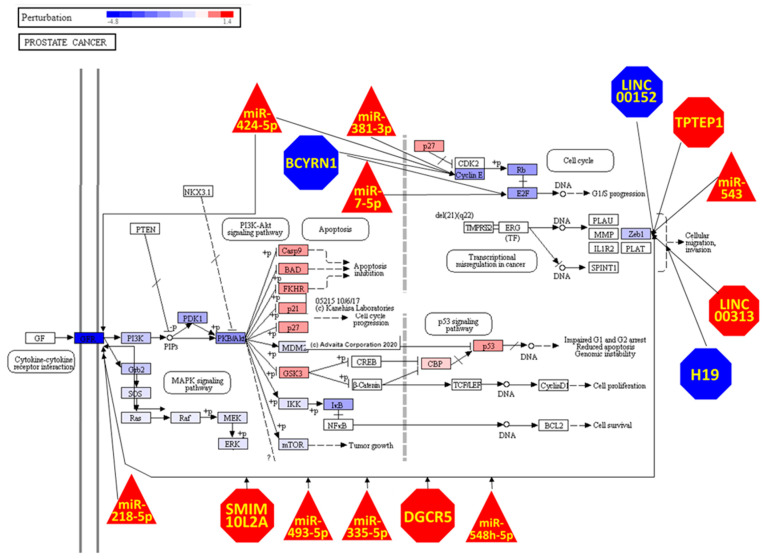
Prostate Cancer KEEG pathway genes being perturbed and regulated by ncRNAs due Cell Senescence. The transcription factor ZEB1 is the target of a large number of ncRNAs. The DE RNA threshold in limma was log 2 Fold Change ≥ |1| and adjusted *p*-value (FDR) < 0.01. The Prostate Cancer KEGG pathway depiction and impact analysis were generated using iPathwayGuide (Advaita). Blue indicates down-regulated and red up-regulated in senescent cells relative to non-senescent cells. Genes are presented as rectangles, miRNAs as triangles, and lncRNAs as octagons.

In addition to its effect on the cell cycle, cell senescence affected the expression of genes linked to DNA repair (Figure 3 and Supplemental Table S2). For the top ncRNAs, only *LINC00152* and *LINC00313* were not linked to a DNA repair gene, which in our model implies that they are specifically linked to the Cell Cycle. On the other hand, the lncRNAs *MYBL*, *SERHL* and *SNHG5* are specifically linked to DNA repair. With *H19*, *DGCR5*, *SMIM10L2A*, *BCYRN1*, *EMX2OS* involved in both processes, miR-335-5p, *BCYRN1* and *H19* interactions seem to represent the common core of both Cell Cycle and DNA repair alterations. *FOXM1* and *CDK1* from the KEGG Senescence pathway (Figure 4) and *EGFR* from the Prostate Cancer Pathway are also involved in the DNA repair process (Figure 5).

### 3.4. Prostate Cell Senescence lnRNAs and miRNAs Are Pivotal in Regulation Cell Senescence and Prostate Cancer KEGG Pathways

Cell Senescence modulated expression of 19 genes belonging to the KEGG Pathway Cell Senescence, and of these 4 (*IL8*, *IGFBP3*, *CDKN2B*, *TRPV4*) are not regulated by any of the top lncRNAs or miRNAs, whereas 15 (*ITPR1*, *CDKN2A*, *NFATC1*, *RBL1*, *SLC25A4*, *MAPK13*, *E2F2*, *CDC25A*, *CCNE2*, *CDK1*, *CCNA2*, *FOXM1*, *CCNB2*, *CCNB1*, *MYBL2*) were regulated by at least one of them (Figure 4). Interestingly, the transcription factor *NFATC1* is the gene being targeted the most by ncRNAs (*TPTEP1, SRHL, miR218-5p* and *miR493-5p*) in this pathway (Figure 4). The lncRNA H19 (the top-ranked lncRNA based on centrality) seems to be regulating *CDKN2A* (represented in Figure 4 as the ARF and p16 proteins), but not *NFATC1*. Consistent with the results described above, the majority of the ncRNA-gene interactions are concentrated in Cell Cycle arrest and the Cell Senescence pathway.

Cell Senescence caused the differential expression of six genes belonging to the KEGG Pathway Prostate Cancer (*PDGFRB*, *PDGFRA*, *CCNE2*, *E2F2*, *EGFR* and *ZEB1*). Impact analysis revealed that the alterations of expression of these genes will perturb the function of several proteins, in particular activation of the phosphatidylinositol 3-kinase (PI3K)/AKT signalling pathway and inhibition of the MAPK signalling pathway (Figure 5). The transcription factor *ZEB1* is the most regulated gene in this pathway being a target of 6 lncRNAs (*LINC00152*, *TPTEP1*, *LINC00313*, *SMIM10L2A*, *DGCR5*) and 4 miRNAs (miR-543, miR-548h-5p, miR-335-5p, miR-493-5p) and it is interesting to note that ncRNA regulation links the extremes of the pathway with the *PDGFRB*, *PDGFRA*, *EGFR* receptors (represented in Figure 5 as GFR) and regulated by 5 ncRNAs that also regulate ZEB1 (*SMIM10L2A*, *DGCR5*, *miR-548h-5p*, *miR-335-5p*, *miR-493-5p*).

## 4. Discussion

We demonstrated that cells in the late passage have an altered transcriptomic profile promoting cell cycle arrest. We observed perturbation in both the PI3K/AKT and EGFR/ERK/p53 pathways. AKT promotes rapid proliferative arrest in the absence of DNA damage or a hyperproliferative phase, suggesting that inactivation of the senescence response is critical at the early stages of PI3K/AKT-driven tumourigenesis. mTORC1 is an essential mediator of AKT-induced senescence [36]. Sophisticated regulation of PI3K/AKT/mTORC1 signalling is necessary for homeostatic control of cell growth, proliferation, and survival. Aberrant activation of this signalling network is an initial driver of many sporadic human cancers. Ironically, continuous hyperactivation of the PI3K/AKT/mTORC1 pathway in nontransformed cells results in cellular senescence, which is a tumour-suppressive mechanism that must be disabled to promote malignant transformation [37]. The PI3K/AKT pathway plays an important role in the senescence and self-renewal of human skin-derived precursors [38]. Pro-inflammatory cytokines namely IL-1β, IL-13, MCP-2, MIP-3α, and SDF-1α) induce cellular senescence through activation of the EGFR-Ras signalling pathway [39]. Furthermore, p53-mediated up-regulation of MKP-3 contributes to the establishment of the senescent cellular phenotype through dephosphorylating ERK1/2 [40]. As there is considerable cross-talk between the PI3K/AKT and EGFR/ERK/p53 pathways regarding their regulation and function, this leads to the hypothesis that inhibiting some of their key proteins could mitigate cellular ageing [41].

miRNAs are reported as markers for both cancer diagnostic and prognostic [42,43,44] and five of our top DE miRNAs (*miR-7*, *miR-335*, *miR-381*, *miR-424*, *miR-543*) are considered potential biomarkers for human prostate cancer [45], a disease that is known to increase in incidence with advanced age [46]. The expression of *miR-335* was found to be increased in senescent human mesenchymal stem cells, rat kidneys and mice brains [47,48,49], and its activity seems to alter cholesterol metabolism and impair mitochondrial function [48,49]. The role of *miR-335* in cancer is contextual; its acts as a tumour promoter on several meningiomas promoting cell proliferation and inhibiting cell cycle arrest [50], but *miR-335* up-regulation was shown to inhibit breast cancer tumorigenesis [51]. It has been recognised as a tumour suppressor for prostate cancer [52] and was shown to restore and increase chemotherapy sensitivity in breast and ovarian cancers [53,54]. *miR-543* was found to regulate human mesenchymal stem cell ageing and is dysregulated in senescent primary mouse embryonic fibroblasts, being down-regulated in both models [55,56]. As with *miR-335*, *miR-543* promotes cell proliferation and invasion in prostate, colorectal and nasopharyngeal cancer [57,58,59], but acts as a tumour suppressor in gliomas [60], and was shown to inhibit tumour growth and metastasis in cervical cancer [61]. In our model, we found up-regulation of *miR-543* in correlation with down-regulation of cell cycle genes, indicative that overexpression of this miRNA is a sign of senescence in prostate cells.

The literature provides little information about the roles of the remaining top miRNAs in the senescence process. Nonetheless, senescence and cancer were shown to have shared characteristics [2,62,63] and in the case of cancer, contextual roles are described for *miR-424-5p* [64,65], *miR-665* [66,67], *miR-484* [68,69] and *miR-7-5p* [70,71] as both tumour promoters and suppressors. Others are described only as tumour suppressors, namely *miR-493-5p* [72,73], *miR-381-3p* [74,75] and *miR-218-5p* [76,77]. No information was found on functional roles for *miR-548h-5p*. Interestingly, *miR-335* and *miR-543* in combination was found to suppress bone metastasis in prostate cancer [78], and a combination of different miRNAs were shown to predict response to radiation therapy [79,80,81,82], giving weight to the notion that it is the combination of miRNAs that regulates the senescent phenotype.

The expression of the lncRNA *H19* was shown to regulate ageing in the endothelial HUVECS cells line [83], and to contribute to oxidative damage repair in age-related cataracts [84]. It’s down-regulation in our cell senescence model is in agreement with the roles described in the literature. In the cellular senescence pathway, the principal targets of *H19* are p16 and ARF, two tumour suppressors molecules, biomarkers of ageing [85] that are up-regulated in our study. The role of *H19* in prostate carcinogenesis and tumour growth has yet to be fully elucidated and, as with the miRNAs, it has been described as both an oncogene and tumour suppressor depending on the context [86,87], although it’s up-regulation is correlated with increased metastasis in prostate cancer [87]. There is a lack of information surrounding the function of the lncRNA *DGCR5*. Recently its role in decreasing stemness in prostate cancer was uncovered [88]. *DGCR5* is strongly related to stem cell properties, both for promoting differentiation and inducing stem cell-like status [89,90], which could influence positively or negatively cancer initiation and progression.

The exact physiological role of *SMIM10L2A* remains to be determined. It encodes a highly conserved small protein that contains a conserved motif (DUF4560) and may function as an integral membrane protein. *SMIM10L2A* associates with enhancer chromatin and therefore may also function as a lnRNA to regulate enhancers. Its increased expression in human gastric cancer correlated positively with overall patient survival [91].

*TPTEP1* expression was shown to inhibit hepatocellular carcinoma progression and non-small cell lung cancer proliferation [92,93]. *BCYRN1* was reported to promote prostate cancer progression [94], and it is described as solely an oncogene [95], which is also the case of *LINC00152* [96], *SNHG5* [97] and *LINC00313* [98,99]. *EMX2OS* was shown to promote proliferation, invasion and stemness in ovarian cancer cells [100], but down-regulation of *EMX2OS* is correlated with a shorter recurrence-free survival in classic papillary thyroid cancer [101]. *SERHL* is hypothesised to be involved in peroxisome function [102] and was shown to be part of a lncRNA signature that predicts recurrence-free survival in hepatocellular carcinoma [103].

The network depicted in Figure 3 shows that senescence affects both cell signalling and cell structure linked to the Cell Cycle. Cell cycle arrest is an early signal of cellular senescence [104] and centrosome abnormalities can cause mitotic errors and genomic instability, accelerating the ageing process and culminating in tumorigenesis [105]. The DNA damage theory of ageing stipulates that DNA damage accumulation is the principal cause of ageing, and defects in DNA repair and processing are linked to accelerated ageing [106]. The effect of miRNAs on the DNA repair machinery is well described [107] and mounting evidence demonstrated that lncRNAs also play an important role in this biological process [108].

The reported ncRNA-target network affected gene expression in both the Cell Senescence and Prostate Cancer pathways. For Cell Senescence, not surprisingly, the ncRNAs interactions are concentrated on the cell cycle axis, with *BCYRN1* and *miR-381-3p* possessing the largest number of interactions (4 each), suggesting that their role is to specifically promote cellular senescence. The gene *NFATC1* is the most regulated gene in this pathway. *NFATC1* up-regulation promotes ageing in mice hair follicles and human fibroblasts [109,110] and contributes to human prostate tumorigenesis [111].

Interestingly, *miR-493-5p* targeted both *NFATC1* and *CCNA2* for silencing, linking two branches of the cell senescence pathway not described by KEGG; considering that this miRNA is normally found suppressed in prostate cancer [112], it makes *miR-493-5p* a good potential therapeutic target for prostate cancer treatment. Its role in cell senescence warrants further investigation.

Although we found only 6 DE genes on the KEGG Prostate Cancer Pathway (*PRGFRA*, *PRGFRB*, *CCNE2*, *E2F2*, *EGFR* and *ZEB1*), impact analysis identified that the down-regulation of all these genes led to EGFR inhibiting the activity of the MAPK signalling pathway but activating the PI3K-Akt and the p53 pathways, also related to senescence [113,114]. There is a strong regulation by ncRNA of *ZEB1*, *PDGFRA*, *PDGFRB* and *EGFR*, connecting the input to the output of the pathway, a mechanism that has not been described previously. The transcription factor *ZEB1* is pivotal in cancer aggressiveness, inducing a stem-like phenotype in cancer cells and invasive behaviour [115], and although the gene plays a support role in tumorigenesis, its expression was not reported to be carcinogenic *per se* [115,116]. Nonetheless, *ZEB1* was revealed to be crucial to maintain and regulate adult stem cells in tissue homeostasis and regeneration [117,118], and stem cell exhaustion is one of the hallmarks of ageing [119], indicating that down-regulation of ZEB1 is linked to cell senescence. The activation of the cell surface protein kinases *PDGFRA*, *PDGFRB* and *EGFR* promotes cell proliferation in normal cells and is enhanced in certain tumour types [120,121], while their down-regulation is in accord with the cell cycle inhibition we uncovered. In conclusion, we report here a miRNA/lncRNA network regulating both DNA repair and cell cycle in primary prostate cell senescence. Future studies will focus on the dual effects of the miRNAs and lncRNAs described here for their possible actions as drug targets and/or therapeutic molecules against ageing and carcinogenesis.

## 5. Conclusions

We described the central role of ncRNAs in the senescence process of primary prostate cells. We showed that senescence induced the perturbation of regulatory ncRNA-gene network, leading to the down-regulation of several genes involved in cell cycle and DNA repair processes, two hallmarks of cellular ageing. Our results put lncRNAs at the centre of this deregulation and describe functions that have not yet been extensively studied. This study opens interesting possibilities for the development of new biomarkers and therapeutics in the treatment of ageing and cancer.

## Figures and Tables

**Table 1:** Prostate Cell Senescence Top 10 Affected Gene Ontology Biological Process terms. Adjusted *p*-value (FDR) < 0.05.

GO: Biological Process	Count DE	GO Size	% Affected	Adj *p*-Value
nuclear division	81	254	31.9%	2.81 × 10^−14^
mitotic cell cycle process	153	661	23.1%	2.81 × 10^−14^
cell cycle process	199	967	20.6%	1.2 × 10^−13^
organelle fission	84	281	29.9%	1.43 × 10^−13^
cell cycle	248	1305	19.0%	1.61 × 10^−13^
sister chromatid segregation	51	128	39.8%	3.35 × 10^−13^
mitotic cell cycle	163	750	21.7%	3.44 × 10^−13^
chromosome segregation	68	210	32.4%	8.33 × 10^−13^
nuclear chromosome segregation	55	158	34.8%	1.43 × 10^−11^
cell division	112	466	24.0%	1.53 × 10^−11^

## Data Availability

The data that support the findings of this study are available at the National Center for Biotechnology Information (NCBI) Gene Expression Omnibus (GEO) database; accession number GSE189209, https://www.ncbi.nlm.nih.gov/geo/query/acc.cgi?acc=GSE189209, accessed on 3 December 2021.

## Supplementary Materials

The following are available online at https://www.mdpi.com/article/10.3390/genes13020208/s1, Table S1: DE analysis Results Prostate Cells. Table S2: GO PB Prostate Cells. Table S3: miRNA DE analysis Results Prostate Cells. Table S4: Micro_RNA_impact. Table S5: DE lncRNAs. Table S6: lncRNA_impact. Table S7: Reg Network. Table S8: GO CC.
